# Health risk assessments of arsenic and toxic heavy metal exposure in drinking water in northeast Iran

**DOI:** 10.1186/s12199-019-0812-x

**Published:** 2019-09-14

**Authors:** Hosein Alidadi, Seyedeh Belin Tavakoly Sany, Batoul Zarif Garaati Oftadeh, Tafaghodi Mohamad, Hosein Shamszade, Maryam Fakhari

**Affiliations:** 1 Department of Environmental Health Engineering, Faculty of Health, Mashhad University of Medical Science, Mashhad, Iran; 2 Department of Health Education and Health Promotion, Faculty of Health, Mashhad University of Medical Sciences, Mashhad, Iran; 3 Students Research Committee, Mashhad University of Medical Sciences, Mashhad, Iran; 4 Social Determinants of Health Research Center, Mashhad University of Medical Sciences, Mashhad, Iran; 5 Chemistry Department, Islamic Azad University of Mashhad, Mashhad, Iran; 6 Statistics Department, Khorasan Razavi Regional Water Authority, Mashhad, Iran; 7 Khorasan Razavi Regional Water Authority, Mashhad, Iran

**Keywords:** Human health risk assessment, Heavy metal pollution, Arsenic, Carcinogenic and non-carcinogenic effects

## Abstract

**Background:**

Arsenic and heavy metals are the main cause of water pollution and impact human health worldwide. Therefore, this study aims to assess the probable health risk (non-carcinogenic and carcinogenic risk) for adults and children that are exposed to arsenic and toxic heavy metals (Pb, Ni, Cr, and Hg) through ingestion and dermal contact with drinking water.

**Method:**

In this study, chemical analysis and testing were conducted on 140 water samples taken from treated drinking water in Mashhad, Iran. The health risk assessments were evaluated using hazard quotient (HQ), hazard index (HI), and lifetime cancer risk (CR).

**Results:**

The results of the HQ values of arsenic and heavy metals for combined pathways were below the safety level (HQ < 1) for adults, while the HI for children were higher than the safety limit in some stations. Likewise, Cr showed the highest average contribution of HI_total elements_ (55 to 71.2%) for adult and children population. The average values of total carcinogenic risk (TCR) through exposure to drinking water for children and adults were 1.33 × 10^−4^ and 7.38 × 10^−5^, respectively.

**Conclusion:**

Overall, the CR_total_ through exposure to drinking water for children and adults was borderline or higher than the safety level of US EPA risk, suggesting the probability of carcinogenic risk for the children and adults to the carcinogenic elements via ingestion and dermal routes. Therefore, appropriate purification improvement programs and control measures should be implemented to protect the health of the residents in this metropolitan city.

**Electronic supplementary material:**

The online version of this article (10.1186/s12199-019-0812-x) contains supplementary material, which is available to authorized users.

## Introduction

Heavy metals and arsenic contamination in drinking water poses a serious threat to human life because of their toxicity, bio-accumulative nature, and persistence in the environment [1–3]. The heavy metals contaminate the groundwater and surface water through a natural process and anthropogenic activities (e.g., industrial, agricultural, mining, and traffic activities) [4, 5]. According to the World Health Organization (WHO) report 2015, 71% of the global population uses safely managed drinking water sources [6]. This includes piped treated water that is located on premises and protected dug wells [7, 8]. However, safely managed water sources can still be polluted by toxic elements due to the poor domestic treatment system, use of chemical materials in the water treatment system, pipeline corrosion, leaching of elements from pipes of water distribution, and use of improper storage containers and poorly maintained filtration for drinking water at home [9, 10]. To date, most of the developing countries are faced with this challenge, usually due to their limited economic capacities to use advanced technologies for heavy metal removal [6].

The greatest threat of toxic heavy metals and arsenic is reported in the drinking water and groundwater of several countries, including Mexico, Saudi Arabia, India, Bangladesh, China, Chile, Thailand, and Iran [1, 7]. In Sonora, Mexico, approximately 43% of a drinking water sample from storage tanks and wells were observed to have elevated levels of Cd, As, Hg, Cu, and Pb [7]. The concentrations of Cd, Pb, and Cu in drinking water in ten cities of Saudi Arabia exceeded the guideline value, which was attributed to the Kuwaiti and the Gulf War oil fires [11]. The concentrations of Mn, Cd, and Pb in drinking water in India exceeded the guideline value, which was attributed to the geo-genic contamination [12]. In the last 10 years, data on heavy metal contamination of groundwater in most rural areas of India showed that the average concentrations of As, Mn, Cr, Pb, Ni, and Zn in drinking water exceeded the WHO guidelines, which was linked to the pharmaceutical, paint, pesticide and fertilizer industries [11, 12]. In the last 14 years, data on As contamination in Bangladesh showed that 42.1% of the drinking samples had As above 50 μg/L [9, 13]. The average concentrations of Pb, Cr, Ni, and Zn in drinking water in some metropolitan cities of Iran [14–16] and Thailand [4, 17] exceeded the guidelines value, which was linked to the pipeline corrosion and poor domestic treatment.

Several studies have evaluated the level of toxic metals in drinking water and reported that the concentrations of these metals from Germany, USA, Jordan, Malaysia, and Turkey are below permissible limits [7, 9, 18]. However, comparisons with standards alone are not enough to quantitatively assess the health risk of toxic element exposure via consumption of drinking water. Human health risk assessments models are recently implemented to examine whether exposure to toxic elements could increase the incidence of adverse effect on human health [19–22].

Studies showed that population growth, increasing water scarcity, urbanization, and climate change are great challenges for drinking water supply systems. By 2025, more than 50% of the global population will be living in water-stressed regions, particularly low- and middle-income countries [7]. Therefore, a determination of the level of heavy metals in different water sources is important for proper human health risk assessment [7, 9]. According to the Environmental Protection Agency (EPA) and Agency for Research on Cancer (IARC), exposure to inorganic arsenic and toxic heavy metals are of major concern in drinking water, mainly due to their carcinogenic and non-carcinogenic effects on human health. Arsenic, Cd, and Cr in drinking water have been pointed as a public health concern in > 30 countries. It was evidenced that drinking 1 L/day drinking water with As dose of 50 μg L^−1^ and Cr dose of 8.3–51 over one’s lifetime may cause of the cancer of the lung, liver, bladder, and kidney [11, 23]. It was also found that skin damages and respiratory disorders were increased from an As dose of 0.0012 mg/kg/day through drinking water. Long-term exposure to Cd leads to chronic renal failure, anosmia, anemia, cardiovascular diseases, hypertension, and osteoporosis [11]. Other effects such as anemia from Pb [9], gastrointestinal disorder from Cu [9, 10], kidney and liver damage from Hg, and blood cholesterol [10, 24] and heart diseases from Sb were also reported [9, 10, 24].

In Iran, although, 96 % of the cities have access to safe water supply systems, drinking water supply can still be contaminated by arsenic and heavy metals [15]. They demonstrated that due to the improper waste disposal and pipeline corrosion, there is an increase in the pollution level of water supply, which in turn has led to increasing skin lesion and incidence of cancer in Iran [14, 15]. This work is the first study of arsenic and heavy metal exposure, in which we provide more knowledge about their dispersion pattern and non-carcinogenic and carcinogenic health risk in Mashhad, Iran.

Several studies have been conducted on evaluating the heavy metal contamination in different environmental matrices (e.g., sediment, soil, and foodstuff) [5, 25]; however, no background and updated databases are available on health risk of toxic heavy metals through drinking water consumption in this region.

Available monitoring data on heavy metals from the village areas of Mashhad have shown symptoms of As and toxic heavy metal contamination [14, 15, 26]. With regard to the importance of Mashhad as the second metropolitan city in Iran (religious capital of Iran) and great industrial and tourism center (25 million per years) [27], greater attention needs to be provided regarding reliable heavy metal information in municipal water distribution system. The aims of this study were to (1) examine the concentrations of arsenic and toxic heavy metals (Cr, Hg, Pb, and Ni) in piped treated water to characterize drinking water quality and (2) to estimate the health risks (non-carcinogenic and carcinogenic) for the residents exposed to arsenic and toxic elements through ingestion and dermal contact with piped treated water.

## Materials and methods

### Study area description

**Fig. 1:**
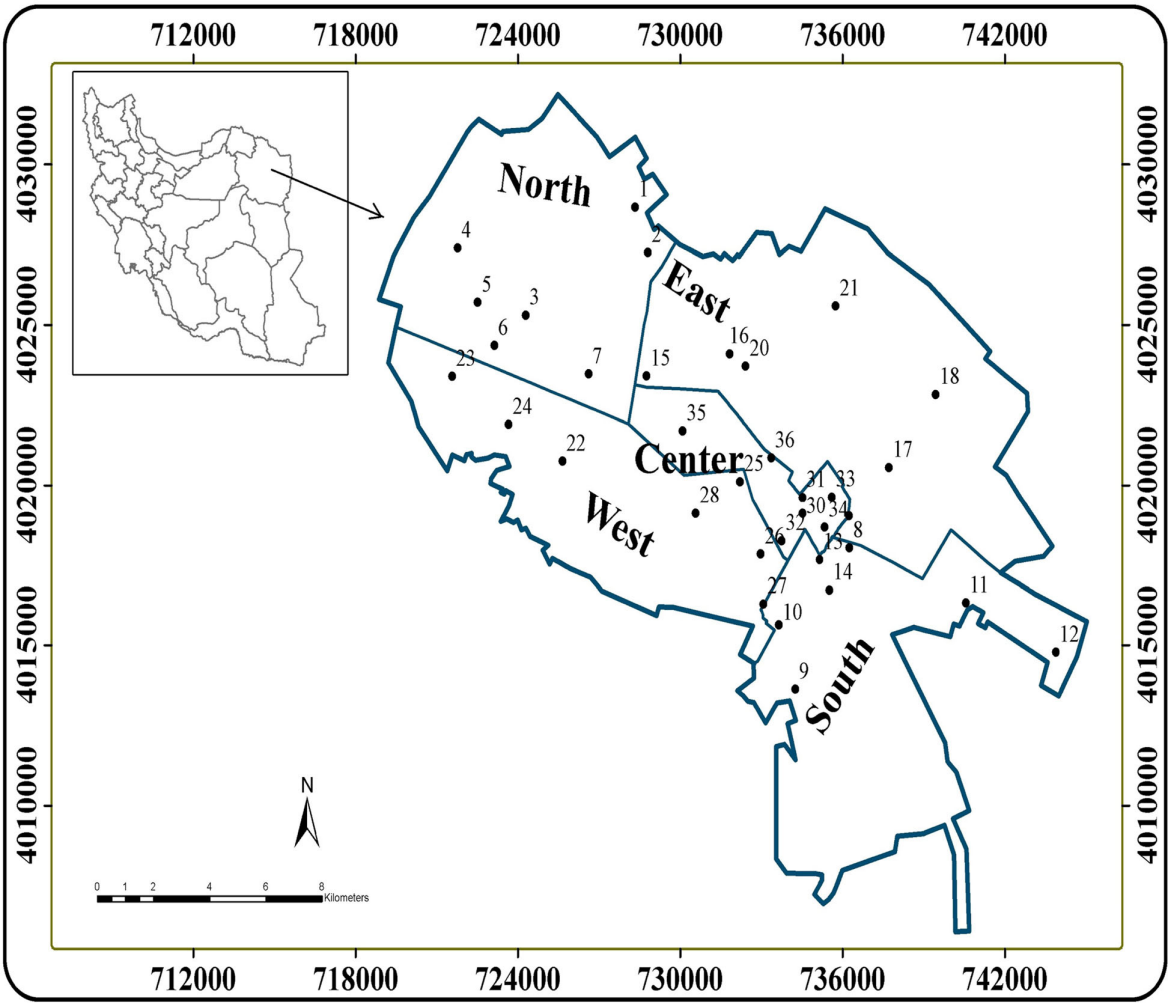
Location of Mashhad, Khorasan Razavi, Iran

Mashhad is the second largest metropolitan city in Iran, which is located in the northeast of Iran, bordering Turkmenistan and Afghanistan between the longitudes of 58° 20′ to 60° 8′ and latitudes 35° 40′ to 36° 3′ of Iran (Fig. 1, Table 1) [27]. This city covers a total area of 16,500 km2, which is limited to the south by Binaloud Mountain, to the north by Hezar Masjid heights, to the southeast by Jamroud river basin, and northwest by Atrak river basin [28, 29]. Mashhad has a population of 3.004 million, of which 97.5% are Persians and 2% (0.054 million) are Pakistani, Afghani, Turkish, and Arab. The region climate is cold and dry [29]. In this city, the supply of drinking water originates from rivers, wells, and groundwater. The conventional water treatment system was used to treat drinking water in Mashhad, and it is distributed through a pipeline system consisting of high-density polyethylene (HDPE) pipes and mild steel [28, 29]. The present study area has suffered several environmental pollutants due to the fastest-growing industrial zones (leather goods, metal products, dyeing, fertilizers, textile, chemical, and so on) and economic development coupled with agricultural activities [5, 29]. The wastewater from agricultural and industrial zones is discharged into the natural rivers and dam and eventually permeates through the groundwater that is used for drinking purpose. This results in a rapid release of heavy metals and other chemical toxins into water sources and subsequently to the human body via the food chain [27, 29]. Furthermore, a large part of the pipeline construction in the present study area is old [29]. Therefore, pipeline corrosion may affect the concentration of heavy metals in the municipal water distribution system.

**Table 1:** Characteristics of sampling stations and statistics of heavy metal concentrations (μg L^−1^)

Sites	Stations	Latitude	Longitude	As	Hg	Pb	Cr	Ni
North	1	4029710	727406	0.15	ND	0.83	0.415	1.22
	2	4027701	728482	0.14	ND	0.415	0.565	0.9
	3	4025335	724454	0.12	ND	0.705	0.97	3.2
	4	4027198	721742	0.27	ND	1.065	6.03	1.225
	5	4025914	725243	0.15	ND	0.485	2.725	1.555
	6	4018132	735467	0.25	ND	0.705	3.65	1.885
	7	4018221	735984	0.20	ND	0.365	17.67	0.45
South	8	4017602	736047	0.17	ND	0.36	0.36	0.795
	9	4014001	733598	0.09	ND	0.525	0.535	0.395
	10	4014963	733130	0.20	ND	0.485	3.825	1.09
	11	4016330	740638	0.21	ND	0.385	8.245	0.185
	12	4014951	743628	0.18	ND	0.31	8.265	1.27
	13	4023448	728776	0.14	ND	0.59	6.41	1.31
	14	4024096	731871	0.18	ND	0.395	10.21	0.36
East	15	4020529	737745	0.10	ND	0.33	0.68	0.28
	16	4022731	739408	0.19	ND	0.515	11.17	0.23
	17	4023714	732412	0.12	ND	0.375	23.25	1.715
	18	4025587	735752	0.28	ND	0.625	0.47	1.815
	19	4025452	734910	0.21	ND	0.495	0.565	0.35
	20	402041	728081	0.15	ND	0.56	0.59	0.58
	21	4023401	721575	0.16	ND	0.75	4.16	0.86
West	22	4024240	723377	0.16	0.2	0.54	2.415	1.715
	23	4021934	723688	0.39	ND	1.33	6.34	13.145
	24	4023475	726604	0.18	ND	0.31	7.51	0.4
	25	4021665	730176	0.18	ND	0.625	0.315	3.05
	26	4020791	733368	0.23	ND	0.53	1.185	2.96
	27	4019883	732363	0.24	ND	0.64	4.455	1.405
	28	4017836	732942	0.20	ND	0.745	5.56	2.47
Center	29	4016239	733048	0.23	ND	0.505	4.08	1.49
	30	4019093	730563	0.20	ND	0.54	2.525	1.145
	31	4019123	734946	0.14	ND	0.56	1.59	1.515
	32	4017372	734784	0.16	0.2	0.575	7.875	0.95
	33	4019664	734467	0.13	ND	0.45	1.435	1.59
	34	4018134	734290	0.20	0.1	0.81	19.5	3.63
	35	4019632	735625	0.14	ND	0.635	0.4	1.19
Mean				0.18	0.01	0.58	4.94	1.69
Standard deviation				0.05	0.048	0.206	5.53	2.15
*Asymp sig				0.01	< 0.001	0.02	< 0.001	0.03
RfD via ingestion pathway (mg/kg/day)				0.0003	0.0003	0.0035	0.003	0.02
RfD via dermal pathway (mg/kg/day)				0.000285	0.0003	0.000525	0.000075	0.0003

*Asymptotic significant at *p* < 0.05 based on Kruskal–Wallis test and testing significant change on the spatial variation of arsenic and toxic elements in drinking water from 35 stations

### Sampling, preservation, and transportation

On the basis of location and land uses, five sites (north, south, east, west, and center) having 35 stations were selected for drinking water sampling. All stations were sampled over four times, twice each in the dry (August and September 2017) and rainy (March and April 2017) seasons. We used cleaned plastic bottles pre-washed with double distilled water and 20% HNO^3^ to collect water samples [30]. The samples were filtered using a 0.45-mm Whatman pore membrane and acidified with 3 ml nitric acid (HNO^3^, 69%) to prevent adsorption and crystallization of trace element prior to further analysis. Then, water samples were transported in cool and dark containers and stored in a refrigerator at 4 °C until laboratory analysis [30].

### Chemical analysis

All the acids, reagents, and standard solution (standard stock solutions, internal standard solutions, and a multi-element solution) were purchased from Merck (Darmstadt, Germany). All filtered and acidified drinking water samples were analyzed for As and toxic heavy metals (Hg, Ni, Cr, and Pb) by using inductively coupled plasma mass spectrometry (ICP-MS, 7700 series) under EPA method 6020 [9] (Additional file 1: Table S1). We used the blank and standards solutions of metal ions to obtain the calibration graph. The calibration blank (analytic-free media) was used with prepared standards to calibrate the ICP-MS (establishing a “zero” setting) and to confirm the absence of interferences in the analytical signal. The standard solution was made using different concentrations of elements following a range of metal ions based on previous studies and the limit of detection (LOD). The correlation coefficients of calibration lines for each metal were found to be greater than 0.99.

### Quality control and assurance

Glass containers and plastic bottles used during the analysis procedures were acid washed in diluted nitric acid solution (HNO3) for 24 h and rinsed using deionized water. Then, bottles were dried at room temperature and kept sealed. The instrumental LOD for water samples was estimated by a standard procedure. The LOD for each heavy metal ion was As (0.11 μg/L), Cr (0.06), Hg (0.01), Pb (0.15), and Ni (0.22), respectively. In order to check the reproducibility of the analysis, each sample was measured in triplicate. In this study, standard reference solutions with known concentration of the heavy metals (spiked solation) were used as control samples to check the measurement precision. Certified reference materials (CRMs) and standard reference solutions with known concentration of elements are recognized to be an essential tool for assuring the quality and establishing the accuracy of the results for the measurements of heavy metals by ICP-MS [9]. After each batch of ten samples, the control sample was analyzed to check the accuracy of the analysis. Recovery rates for each element were in acceptable ranges (85.7–115%). Accepted recovery ranged from 80 to 120%. All concentrations of As and elements were reported in μg L^−1^ on a fresh weight basis. We used the average concentration of each element for further interpretation because the reproducibility was at 95% confidence level. All these analyses were conducted in the water quality laboratory of Water Authority of Khorasan Razavi Province, Iran.

### Health risk assessment

#### Problem formulation

According to the United States Environment Protection Agency (US EPA), the further intake of As and heavy metals such as Ni, Cr, Pb, and Hg through drinking water may increase the non-carcinogenic and carcinogenic risk on human health [22]. Therefore, in the present study, the first assumption about health risk was that there is a serious carcinogenic or non-carcinogenic risk posed by As and toxic elements via the consumption of drinking water in Mashhad, Iran. Another assumption was that the dermal exposure of As and toxic elements from drinking water contributes to increasing the health risk in the study area. In the recent decade, the US EPA suggests that the human health risk assessment (HHRA) model measures the potential health risk of investigated contaminants using exposure and toxicity determination [19, 22].

#### Exposure assessment

Average daily dose (ADD) was implemented to estimate human exposure dose to arsenic and toxic metals through direct ingestion and dermal absorption pathways using Eqs. (1) and (2), which were adapted from the US EPA 2004 and 2005 [19, 22]. Estimations were conducted for two groups; children (as a sensitive group) and adults (as the general population), separately.

1 $$ {\mathrm{ADD}}_{\mathrm{ing}}=\left(\mathrm{C}\times {\mathrm{IR}}_{\mathrm{d}}\times \mathrm{EF}\times \mathrm{ED}\right)/\mathrm{BW}\times \mathrm{AT} $$

2 $$ {\mathrm{ADD}}_{\mathrm{derm}}=\left(\mathrm{C}\times \mathrm{SA}\times \mathrm{SL}\times \mathrm{ABS}\times \mathrm{EF}\times \mathrm{ED}\right)/\mathrm{BW}\times \mathrm{AT} $$

where ADD is expressed as average daily dose of elements through ingestion pathways (ADD_ing_) and dermal absorption (ADD_derm_) (μg kg^−1^ day^−1^), C is the concentration of the heavy metals (μg L^−1^), IR_d_ is the daily ingestion rate (L day^−1^), and its average consumption rates for Iranian children and adults is 1.8 and 2 l per day [31], respectively. The body weight (BW) of child and adult groups is 16 and 70 kg, respectively. ED expressed as the duration of human exposure for children and adults is 6 and 30 years [31], EF is exposure frequency (365 days year^−1^), and AT is averaging time of human exposure, At = 70 × 365 for carcinogenic and AT = ED × 365 days for non-carcinogenic. SL, the skin adherence factor, for children and adults was 200 and 70 (μg cm^2^ h^−1^), respectively. SA is skin surface area for contact with water for children (2800 cm^2^) and adults (5700 cm^2^), and ABS is the dermal absorption factor, ABS = 0.01 for carcinogenic, and ABS = 0.001 for non-carcinogenic.

#### Non-carcinogenic risks

We used Eqs. (3) and (4) to estimate the non-carcinogenic risks using the target hazard quotient (THQ) and hazard index (HI) [22]. THQ is the ratio between the reference dose (RfD) and ADD of each element. In this study, the RfD of each element was adopted from US EPA screening levels [32]. The exposed population is assumed to be safe when HQ lower than 1 [19, 22].

3 $$ \mathrm{THQ}=\mathrm{CDI}/\mathrm{RfD} $$

4 $$ \mathrm{Total}\ \mathrm{THQ}\ \left(\mathrm{HI}\right)=\sum \mathrm{THQ} $$

where RfD is the oral reference dose (μg kg^−1^ day^−1^) that indicate “the daily exposure to which the human population could be continually exposed over a lifetime without an appreciable risk of deleterious effects.” We also estimated HI to measure the total non-carcinogenic risks from different exposure pathways [22].

#### Carcinogenic risks

EPA defined carcinogenic or cancer risks (CR) as “the incremental probability of an individual to develop cancer, over a lifetime, as a result of exposure to a potential carcinogen” [33]. We used Eq. (5) to estimate the carcinogenic risks. The cancer slope factor (CSF) value (μg kg^−1^day^−1^) is only available for As, Pb, and Cr [10, 32], which were adopted from US EPA screening levels [32]. A risk level of 1 × 10^−6^ has been considered as the point of excess cancer risk, indicating 1 per 1,000,000 chance of getting cancer via consumption of drinking water containing arsenic and toxic metals, estimated in μg L^−1^ for 70 years. The safe point for carcinogenic risks must be lower than this level [10]. The range of risks borderline by the EPA is 1 × 10^−4^ to 1 × 10^−6^ and unacceptable if the risks are surpassing 1 × 10^−4^. A carcinogenic risk of 1 × 10^−4^ poses health hazards; therefore, it is sufficiently large, poses health hazards, and need some sort of intervention and remediation [9].

5 $$ \mathrm{CR}=\mathrm{CDI}\times \mathrm{CSF} $$

### Statistical analysis

We used SPSS 17 (SPSS, Chicago, IL) and statistical software Excel 2007 (Microsoft Office) to calculate descriptive statistics. The bivariate analysis was performed to examine the significant variation of heavy metal in different groups.

## Results and discussion

### Heavy metal distribution

All information on the spatial variation of arsenic and toxic elements in drinking water from 35 stations are presented in Table 1. The average concentration of As and other heavy metals in drinking water were significantly different either at stations (*P* < 0.05, df = 25, sig < 0.001) or in sites (*P* < 0.05, df = 4, sig = 0.00). The high concentrations of As, Ni, Hg, and Pb were measured in the west site (stations 23, 24, and 22) and north site (stations 4 and 6). The highest Cr concentration in water samples was measured in the center site (stations 32 and 34) and east site at the station 17. Studies elsewhere showed that several factors such as source water, pipeline corrosion, poor purification system, and water dynamics affect the concentration of heavy metals in pipeline drinking water [34, 35].

In this study, the water samples collected from the west part originated from groundwater and wells, and the high concentration of As, Ni, and Pb may be due to the concentration of natural metals in these sources. The geochemical studies conducted in the west of Mashhad proposed two different sources of heavy metals, which include the ophiolite rocks as the origin of V, Ni, Pb, and Fe and acidic rocks as the origin of Cd, Cu, and As [36, 37]. Therefore, these heavy metals from ophiolite rocks and acidic rocks could be released into groundwater supplies and eventually contaminate these sources in the west region. Furthermore, it has been reported that heavy metals and As are the main pollutants of groundwater supplies in Mashhad [26, 29].

Likewise, the water samples collected from the center and eastern regions originated from Doosti dam water (at the border of Turkmenistan and Iran), and the high concentration of Hg and Cr might be related to the water quality of this dam. The recent studies showed that water in the Doosti dam is contaminated by Cr and Hg [37].

Peiravi et al. (2013) reported that the heavy metals are released into the Doosti dam through the industrial and agricultural processes in areas of land around Doosti dam. It was evidenced that the industrial and agricultural activities generate wastewaters, which are mostly discharged into this water supply. Industrial activities, especially plastic, chemical industries, and metal smelting, are major sources of heavy metals in water. These industries do not use advanced technologies for heavy metal removal, usually due to their limited economic capacities [37, 27]. Furthermore, it was evidenced that pipeline transport in the center part of Mashhad was old [38]. This may affect the drinking water quality in this region. Therefore, suitable sanitation improvement programs should be used to protect the health of the residents in this area.

In an attempt at a rough comparison, heavy metal concentration was compared to other studies in Iran and other countries (Table 2). The average concentration of As, Pb, and Hg found in the drinking water from all sites were lower than that reported for drinking water from Ahvaz and Tehran [14, 16]. In terms of Cr and Ni, there is still no report for drinking water in Tehran and Ahvaz as metropolitan cities in Iran [14, 16]. The average of Cr content in drinking water in this study was lower in India [12], while its concentration was higher in Australia, China, Thailand, and Malaysia [1, 4, 10]. For Pb, Hg, and Ni, the average content in drinking water was lower in Australia, China, Thailand, India, and Malaysia [1, 4, 10]. We found that the average concentration of As was lower than that reported values of drinking water from Australia, Thailand, India, and Malaysia. Likewise, the concentration of arsenic and heavy metals were compared with permissible levels set by the Iranian Ministry of Health, EPA, and WHO [2, 10]. Generally, the relative concentration of As and other heavy metals in this study were far below the permissible limits to assure safe consumption of drinking water (Table 2).

**Table 2:** Arsenic and heavy metal concentrations (μg L^−1^) of drinking water samples collected in this study and comparison of elemental concentrations with other studies and water standard

Heavy metals	As	Hg	Pb	Cr	Ni
In this study (*n* = 140)					
North	0.18 ± 0.058	NA	0.65 ± 0.25	4. 58 ± 6.11	1.49 ± 0.88
South	0.17 ± 0.042	NA	0.65 ± 0.99	5.41 ± 3.91	0.77 ± 0.46
East	0.17 ± 0.060	NA	0.52 ± 0.12	5.84 ± 8.61	0.83 ± 0.67
West	0.23 ± 0.08	0.03 ± 0.08	0.67 ± 0.32	3.98 ± 2.72	3.59 ± 4.31
Center	0.17 ± 0.045	0.04 ± 0.02	0.60 ± 0.12	4.91 ± 6.33	1.79 ± 0.915
Other cities					
Ahvaz* [1]	5.80 ± 1.63	2.8 ± 0.3	21.1 ± 4.4	5.3 ± 3.6	–
Tehran** [2]	2.3 ± 0.76	0.52 ± 0.03	4.5 ± 0.49	–	–
Other countries					
Australia [3]	0.64 ± 0.1	–	5.21 ± 1.43	4.43 ± 1.21	7.93 ± 2.4
China [4]	–	0.07 ± 0.25	5.06 ± 1.73	2.84 ± 0.76	–
Thailand [5, 6]	1.06 ± 1.74	0.10 ± 0.13	16.7 ± 18.5	0.58 ± 0.52	6.13 ± 4.38
India [7]	32 ± 5.6	0.76 ± 0.32	46.2 ± 12.56	28.3 ± 4.9	34.6 ± 14.17
Malaysia [8]	2.51 ± 0.65	0.11 ± 0.0.6	5.18 ± 1.04	2.19 ± 0.93	5.63 ± 1.67
Standards					
WHO [9]	10	6	10	50	70
EPA [6]	10	2	15	100	20
Local standard in Iran^a^ [10]	10	6	10	50	70

*NA* lower then detection limit

*It is a city in the Southwest of Iran and the capital of Khuzestan province

**Capital of Iran

^a^It was set by Iranian Ministry of Health

### Health risk assessment

#### Average daily dose

Exposure to arsenic and heavy metal contaminants through drinking water is a public health concern, and it is important that health risk assessments and impact on environmental health are investigated. Since no information is available regarding the non-carcinogenic effects through arsenic and heavy metals in drinking water of Mashhad, the average daily dose (ADD) of As and four toxic elements were measured through ingestion of drinking water and dermal absorption pathway (dishwashing, swimming, and bathing) (Table 3 and Table 4).

**Table 3:** Descriptive statistics of estimated average daily dose (ADD) of heavy metals for adults and children through drinking water ingestion pathway

Sites/stations (*n* = 140)		ADD of individuals heavy metals (μg kg^−1^ day^−1^)									
		As		Hg		Pb		Cr		Ni	
		Adult	Child	Adult	Child	Adult	Child	Adult	Child	Adult	Child
North	1	0.005	0.017	ND	ND	0.030	0.093	0.015	0.047	0.044	0.137
	2	0.005	0.016	ND	ND	0.015	0.047	0.020	0.064	0.032	0.101
	3	0.004	0.014	ND	ND	0.025	0.079	0.035	0.109	0.114	0.360
	4	0.010	0.030	ND	ND	0.038	0.120	0.215	0.678	0.044	0.138
	5	0.005	0.017	ND	ND	0.017	0.055	0.097	0.307	0.056	0.175
	6	0.009	0.028	ND	ND	0.025	0.079	0.130	0.411	0.067	0.212
	7	0.007	0.023	ND	ND	0.013	0.041	0.631	1.988	0.016	0.051
South	8	0.006	0.019	ND	ND	0.013	0.041	0.013	0.041	0.028	0.089
	9	0.003	0.010	ND	ND	0.019	0.059	0.019	0.060	0.014	0.044
	10	0.007	0.023	ND	ND	0.017	0.055	0.137	0.430	0.039	0.123
	11	0.008	0.024	ND	ND	0.014	0.043	0.294	0.928	0.007	0.021
	12	0.006	0.020	ND	ND	0.011	0.035	0.295	0.930	0.045	0.143
	13	0.005	0.016	ND	ND	0.021	0.066	0.229	0.721	0.047	0.147
	14	0.006	0.020	ND	ND	0.014	0.044	0.365	1.149	0.013	0.041
East	15	0.004	0.011	ND	ND	0.012	0.037	0.024	0.077	0.010	0.032
	16	0.007	0.021	ND	ND	0.018	0.058	0.399	1.257	0.008	0.026
	17	0.004	0.014	ND	ND	0.013	0.042	0.830	2.616	0.061	0.193
	18	0.010	0.032	ND	ND	0.022	0.070	0.017	0.053	0.065	0.204
	19	0.008	0.024	ND	ND	0.018	0.056	0.020	0.064	0.013	0.039
	20	0.005	0.017	ND	ND	0.020	0.063	0.021	0.066	0.021	0.065
	21	0.006	0.018	ND	ND	0.027	0.084	0.149	0.468	0.031	0.097
West	22	0.006	0.018	0.007	0.023	0.019	0.061	0.086	0.272	0.061	0.193
	23	0.014	0.044	ND	ND	0.048	0.150	0.226	0.713	0.469	1.479
	24	0.006	0.020	ND	ND	0.011	0.035	0.268	0.845	0.014	0.045
	25	0.006	0.020	ND	ND	0.022	0.070	0.011	0.035	0.109	0.343
	26	0.008	0.026	ND	ND	0.019	0.060	0.042	0.133	0.106	0.333
	27	0.009	0.027	ND	ND	0.023	0.072	0.159	0.501	0.050	0.158
	28	0.007	0.023	ND	ND	0.027	0.084	0.199	0.626	0.088	0.278
Center	29	0.008	0.026	ND	ND	0.018	0.057	0.146	0.459	0.053	0.168
	30	0.007	0.023	ND	ND	0.019	0.061	0.090	0.284	0.041	0.129
	31	0.005	0.016	ND	ND	0.020	0.063	0.057	0.179	0.054	0.170
	32	0.006	0.018	0.007	0.023	0.021	0.065	0.281	0.886	0.034	0.107
	33	0.005	0.015	ND	ND	0.016	0.051	0.051	0.161	0.057	0.179
	34	0.007	0.023	0.004	0.011	0.029	0.091	0.696	2.194	0.130	0.408
	35	0.005	0.016	ND	ND	0.023	0.071	0.014	0.045	0.043	0.134
Mean		0.007	0.021	0.001	0.002	0.020	0.064	0.180	0.566	0.060	0.187
Standard deviation		0.002	0.006	0.002	0.005	0.007	0.023	0.199	0.628	0.077	0.242

**Table 4:** Descriptive statistics of estimated average daily dose (ADD) of heavy metals for adults and children through dermal intake of drinking water

Sites/stations (*n* = 140)		ADD of individuals heavy metals (μg kg^−1^ day^−1^)									
		As		Hg		Pb		Cr		Ni	
		Adult	Child	Adult	Child	Adult	Child	Adult	Child	Adult	Child
North	1	0.001	0.005	ND	ND	0.006	0.029	0.003	0.015	0.009	0.043
	2	0.001	0.005	ND	ND	0.003	0.015	0.004	0.020	0.006	0.032
	3	0.001	0.004	ND	ND	0.005	0.025	0.007	0.034	0.023	0.112
	4	0.002	0.009	ND	ND	0.008	0.037	0.043	0.211	0.009	0.043
	5	0.001	0.005	ND	ND	0.003	0.017	0.019	0.095	0.011	0.054
	6	0.002	0.009	ND	ND	0.005	0.025	0.026	0.128	0.013	0.066
	7	0.001	0.007	ND	ND	0.003	0.013	0.126	0.618	0.003	0.016
South	8	0.001	0.006	ND	ND	0.003	0.013	0.003	0.013	0.006	0.028
	9	0.001	0.003	ND	ND	0.004	0.018	0.004	0.019	0.003	0.014
	10	0.001	0.007	ND	ND	0.003	0.017	0.027	0.134	0.008	0.038
	11	0.002	0.007	ND	ND	0.003	0.013	0.059	0.289	0.001	0.006
	12	0.001	0.006	ND	ND	0.002	0.011	0.059	0.289	0.009	0.044
	13	0.001	0.005	ND	ND	0.004	0.021	0.046	0.224	0.009	0.046
	14	0.001	0.006	ND	ND	0.003	0.014	0.073	0.357	0.003	0.013
East	15	0.001	0.004	ND	ND	0.002	0.012	0.005	0.024	0.002	0.010
	16	0.001	0.007	ND	ND	0.004	0.018	0.080	0.391	0.002	0.008
	17	0.001	0.004	ND	ND	0.003	0.013	0.166	0.814	0.012	0.060
	18	0.002	0.010	ND	ND	0.004	0.022	0.003	0.016	0.013	0.064
	19	0.002	0.007	ND	ND	0.004	0.017	0.004	0.020	0.002	0.012
	20	0.001	0.005	ND	ND	0.004	0.020	0.004	0.021	0.004	0.020
	21	0.001	0.006	ND	ND	0.005	0.026	0.030	0.146	0.006	0.030
West	22	0.001	0.006	0.001	0.007	0.004	0.019	0.017	0.085	0.012	0.060
	23	0.003	0.014	ND	ND	0.009	0.047	0.045	0.222	0.094	0.460
	24	0.001	0.006	ND	ND	0.002	0.011	0.054	0.263	0.003	0.014
	25	0.001	0.006	ND	ND	0.004	0.022	0.002	0.011	0.022	0.107
	26	0.002	0.008	ND	ND	0.004	0.019	0.008	0.041	0.021	0.104
	27	0.002	0.008	ND	ND	0.005	0.022	0.032	0.156	0.010	0.049
	28	0.001	0.007	ND	ND	0.005	0.026	0.040	0.195	0.018	0.086
Center	29	0.002	0.008	ND	ND	0.004	0.018	0.029	0.143	0.011	0.052
	30	0.001	0.007	ND	ND	0.004	0.019	0.018	0.088	0.008	0.040
	31	0.001	0.005	ND	ND	0.004	0.020	0.011	0.056	0.011	0.053
	32	0.001	0.006	0.001	0.007	0.004	0.020	0.056	0.276	0.007	0.033
	33	0.001	0.005	ND	ND	0.003	0.016	0.010	0.050	0.011	0.056
	34	0.001	0.007	0.001	0.004	0.006	0.028	0.139	0.683	0.026	0.127
	35	0.001	0.005	ND	ND	0.005	0.022	0.003	0.014	0.008	0.042
Mean		0.001	0.006	0.0009	0.001	0.004	0.020	0.036	0.176	0.012	0.058
Standard deviation		0.00021	0.002	0.00083	0.002	0.001	0.007	0.040	0.195	0.015	0.075

**Fig. 2:**
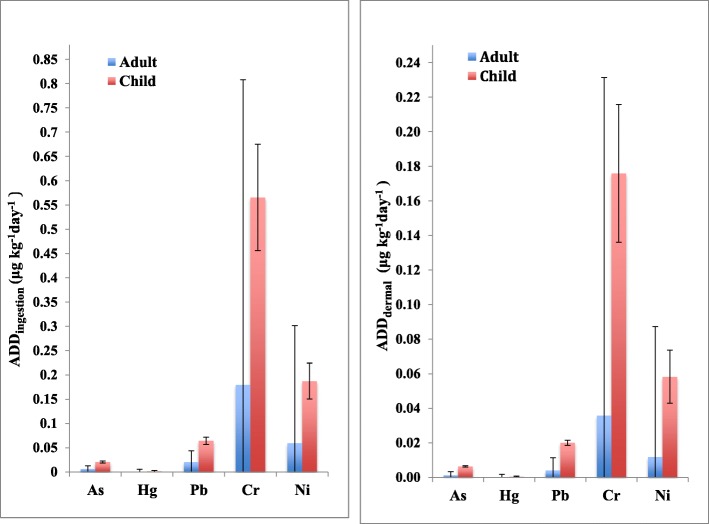
Estimated average daily dose (ADD) for drinking water through ingestion and dermal contact by adult and children

In this study, water ingestion accounted for the majority of ADD_total_ (ADD_ingestion_ + ADD_dermal contact_) of As (2.13%), Hg (0.17%), Pb (6.38%), Cr (63.89%), and Ni (20.80%). The dermal pathway through water contributed a low portion (6.77%) of ADD_total_ for As, Hg, Pb, Cr, and Ni, which accounted for 0.25%, 0.83%, 2.91%, and 2.76 %, respectively. The daily intakes of the Cr and Ni through drinking water ingestion played the most important contribution of ADD_total_ between both target groups. By contrast, the ADD values through the ingestion pathway were ~ 3.2 and 5.5 orders of magnitude higher than the ADD values through dermal absorption pathway (Fig. 2). Therefore, human exposure to arsenic and other toxic metals through water consumption is considered as the important pathways for heavy metal exposure. This result is in agreement with recent studies that reported the most important exposure pathway for heavy metals and arsenic in drinking water occurs through the ingestion route. Furthermore, the mean value of total ADD indicated that children were ~ 3 times more exposed to drinking water than adults (Fig. 2). This result was in accordance with several studies. They reported that the total heavy metal intake doses of children were significantly higher than adults. For instance, in Australia and Thailand, the average ADD_total_ values via the ingestion of drinking water in children populations were ~ 1.7 and 2.5 times higher than adults, respectively [4, 10].

#### Non-carcinogenic risk

**Fig. 3:**
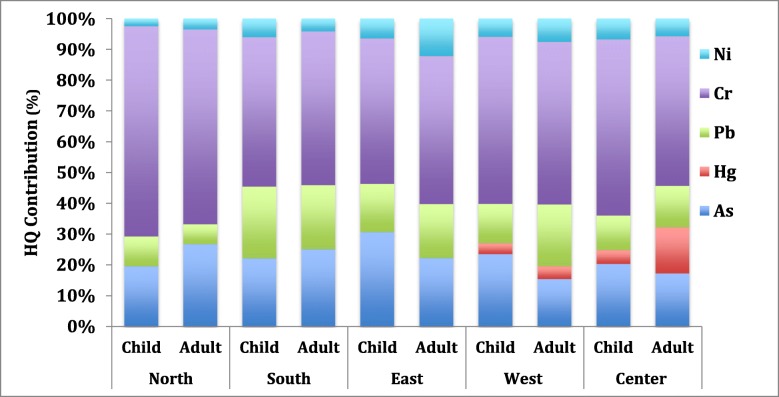
Contribution of input variables on drinking water HI for two age groups

A summary of HQ and HIs values for arsenic and four metals in drinking water through ingestion and dermal contacts with adults and children are presented in Fig. 3, Table 5, and Table 6. As seen from the data, the HQ_ingestion_ and HI_ingestion_ values through ingestion exposure did not exceed the threshold of HQ and HI for adults as well as children.

**Table 5:** Target hazard quotient (THQ) and non-carcinogenic risk (HI) of heavy metals for adults and children through ingestion of drinking water

Sites/stations		THQ of individual heavy metals (*n* = 140)										∑THQ	
		As		Hg		Pb		Cr		Ni		HI	
		Adult	Child	Adult	Child	Adult	Child	Adult	Child	Adult	Child	Adult	Child
North	1	0.018	0.056	0.000	0.000	0.021	0.067	0.005	0.016	0.002	0.007	0.046	0.145
	2	0.017	0.053	0.000	0.000	0.011	0.033	0.007	0.021	0.002	0.005	0.036	0.112
	3	0.014	0.045	0.000	0.000	0.018	0.057	0.012	0.036	0.006	0.018	0.050	0.156
	4	0.032	0.101	0.000	0.000	0.027	0.086	0.072	0.226	0.002	0.007	0.133	0.420
	5	0.018	0.056	0.000	0.000	0.012	0.039	0.032	0.102	0.003	0.009	0.065	0.206
	6	0.030	0.094	0.000	0.000	0.018	0.057	0.043	0.137	0.003	0.011	0.095	0.298
	7	0.024	0.075	0.000	0.000	0.009	0.029	0.210	0.663	0.001	0.003	0.244	0.769
South	8	0.020	0.064	0.000	0.000	0.009	0.029	0.004	0.014	0.001	0.004	0.035	0.111
	9	0.011	0.034	0.000	0.000	0.013	0.042	0.006	0.020	0.001	0.002	0.031	0.098
	10	0.024	0.075	0.000	0.000	0.012	0.039	0.046	0.143	0.002	0.006	0.084	0.264
	11	0.025	0.079	0.000	0.000	0.010	0.031	0.098	0.309	0.000	0.001	0.133	0.420
	12	0.021	0.068	0.000	0.000	0.008	0.025	0.098	0.310	0.002	0.007	0.130	0.409
	13	0.017	0.053	0.000	0.000	0.015	0.047	0.076	0.240	0.002	0.007	0.110	0.348
	14	0.021	0.068	0.000	0.000	0.010	0.032	0.122	0.383	0.001	0.002	0.154	0.484
East	15	0.012	0.038	0.000	0.000	0.008	0.027	0.008	0.026	0.001	0.002	0.029	0.091
	16	0.023	0.071	0.000	0.000	0.013	0.041	0.133	0.419	0.000	0.001	0.169	0.533
	17	0.014	0.045	0.000	0.000	0.010	0.030	0.277	0.872	0.003	0.010	0.304	0.957
	18	0.033	0.105	0.000	0.000	0.016	0.050	0.006	0.018	0.003	0.010	0.058	0.183
	19	0.025	0.079	0.000	0.000	0.013	0.040	0.007	0.021	0.001	0.002	0.045	0.142
	20	0.018	0.056	0.000	0.000	0.014	0.045	0.007	0.022	0.001	0.003	0.040	0.127
	21	0.019	0.060	0.000	0.000	0.019	0.060	0.050	0.156	0.002	0.005	0.089	0.281
West	22	0.019	0.060	0.024	0.075	0.014	0.043	0.029	0.091	0.003	0.010	0.088	0.279
	23	0.046	0.146	0.000	0.000	0.034	0.107	0.075	0.238	0.023	0.074	0.179	0.565
	24	0.021	0.068	0.000	0.000	0.008	0.025	0.089	0.282	0.001	0.002	0.119	0.376
	25	0.021	0.068	0.000	0.000	0.016	0.050	0.004	0.012	0.005	0.017	0.047	0.147
	26	0.027	0.086	0.000	0.000	0.014	0.043	0.014	0.044	0.005	0.017	0.060	0.190
	27	0.029	0.090	0.000	0.000	0.016	0.051	0.053	0.167	0.003	0.008	0.100	0.316
	28	0.024	0.075	0.000	0.000	0.019	0.060	0.066	0.209	0.004	0.014	0.113	0.357
Center	29	0.027	0.086	0.000	0.000	0.013	0.041	0.049	0.153	0.003	0.008	0.091	0.288
	30	0.024	0.075	0.000	0.000	0.014	0.043	0.030	0.095	0.002	0.006	0.070	0.220
	31	0.017	0.053	0.000	0.000	0.014	0.045	0.019	0.060	0.003	0.009	0.053	0.166
	32	0.019	0.060	0.024	0.075	0.015	0.046	0.094	0.295	0.002	0.005	0.153	0.482
	33	0.015	0.049	0.000	0.000	0.011	0.036	0.017	0.054	0.003	0.009	0.047	0.148
	34	0.024	0.075	0.012	0.038	0.021	0.065	0.232	0.731	0.006	0.020	0.295	0.929
	35	0.017	0.053	0.000	0.000	0.016	0.051	0.005	0.015	0.002	0.007	0.040	0.125
Mean		0.022	0.069	0.002	0.005	0.015	0.046	0.060	0.189	0.003	0.009	0.101	0.318
Standard deviation		0.007	0.021	0.006	0.018	0.005	0.017	0.066	0.209	0.004	0.012	0.069	0.219

**Table 6:** Target hazard quotient (THQ) and non-carcinogenic risk (HI) of heavy metals for adults and children through dermal contact of drinking water

Sites/stations		THQ of individual heavy metals (*n* = 140)										∑THQ	
		As		Hg		Pb		Cr		Ni		HI	
		Adult	Child	Adult	Child	Adult	Child	Adult	Child	Adult	Child	Adult	Child
North	1	0.004	0.019	0.000	0.000	0.011	0.055	0.004	0.194	0.011	0.053	0.030	0.321
	2	0.004	0.018	0.000	0.000	0.006	0.028	0.005	0.026	0.008	0.039	0.023	0.111
	3	0.003	0.015	0.000	0.000	0.010	0.047	0.009	0.045	0.029	0.140	0.050	0.247
	4	0.007	0.034	0.000	0.000	0.014	0.071	0.057	0.281	0.011	0.054	0.090	0.440
	5	0.004	0.019	0.000	0.000	0.007	0.032	0.026	0.127	0.014	0.068	0.050	0.246
	6	0.006	0.031	0.000	0.000	0.010	0.047	0.035	0.170	0.017	0.082	0.067	0.331
	7	0.005	0.025	0.000	0.000	0.005	0.024	0.168	0.825	0.004	0.020	0.182	0.894
South	8	0.004	0.021	0.000	0.000	0.005	0.024	0.003	0.017	0.007	0.035	0.020	0.097
	9	0.002	0.011	0.000	0.000	0.007	0.035	0.005	0.025	0.004	0.017	0.018	0.088
	10	0.005	0.025	0.000	0.000	0.007	0.032	0.036	0.179	0.010	0.048	0.058	0.284
	11	0.005	0.026	0.000	0.000	0.005	0.026	0.078	0.385	0.002	0.008	0.091	0.445
	12	0.005	0.023	0.000	0.000	0.004	0.021	0.079	0.386	0.011	0.056	0.099	0.484
	13	0.004	0.018	0.000	0.000	0.008	0.039	0.061	0.299	0.012	0.057	0.084	0.413
	14	0.005	0.023	0.000	0.000	0.005	0.026	0.097	0.476	0.003	0.016	0.110	0.541
East	15	0.003	0.013	0.000	0.000	0.004	0.022	0.006	0.032	0.002	0.012	0.016	0.078
	16	0.005	0.024	0.000	0.000	0.007	0.034	0.106	0.521	0.002	0.010	0.120	0.589
	17	0.003	0.015	0.000	0.000	0.005	0.025	0.221	1.085	0.015	0.075	0.244	1.200
	18	0.007	0.035	0.000	0.000	0.008	0.042	0.004	0.022	0.016	0.079	0.036	0.178
	19	0.005	0.026	0.000	0.000	0.007	0.033	0.005	0.026	0.003	0.015	0.021	0.101
	20	0.004	0.019	0.000	0.000	0.008	0.037	0.006	0.028	0.005	0.025	0.022	0.109
	21	0.004	0.020	0.000	0.000	0.010	0.050	0.040	0.194	0.008	0.038	0.061	0.302
West	22	0.004	0.020	0.005	0.023	0.007	0.036	0.023	0.113	0.015	0.075	0.054	0.267
	23	0.010	0.049	0.000	0.000	0.018	0.089	0.060	0.296	0.117	0.575	0.205	1.008
	24	0.005	0.023	0.000	0.000	0.004	0.021	0.071	0.350	0.004	0.018	0.084	0.411
	25	0.005	0.023	0.000	0.000	0.008	0.042	0.003	0.015	0.027	0.133	0.043	0.212
	26	0.006	0.029	0.000	0.000	0.007	0.035	0.011	0.055	0.026	0.130	0.051	0.249
	27	0.006	0.030	0.000	0.000	0.009	0.043	0.042	0.208	0.013	0.061	0.070	0.342
	28	0.005	0.025	0.000	0.000	0.010	0.050	0.053	0.259	0.022	0.108	0.090	0.442
Center	29	0.006	0.029	0.000	0.000	0.007	0.034	0.039	0.190	0.013	0.065	0.065	0.318
	30	0.005	0.025	0.000	0.000	0.007	0.036	0.024	0.118	0.010	0.050	0.047	0.229
	31	0.004	0.018	0.000	0.000	0.008	0.037	0.015	0.074	0.013	0.066	0.040	0.195
	32	0.004	0.020	0.005	0.023	0.008	0.038	0.075	0.368	0.008	0.042	0.100	0.491
	33	0.003	0.016	0.000	0.000	0.006	0.030	0.014	0.067	0.014	0.070	0.037	0.183
	34	0.005	0.025	0.002	0.012	0.011	0.054	0.185	0.910	0.032	0.159	0.236	1.159
	35	0.004	0.018	0.000	0.000	0.009	0.042	0.004	0.019	0.011	0.052	0.027	0.131
Mean		0.005	0.023	0.000	0.002	0.008	0.038	0.048	0.240	0.015	0.073	0.075	0.375
Standard deviation		0.001	0.007	0.001	0.006	0.003	0.014	0.053	0.258	0.019	0.094	0.059	0.285

This suggested that the daily intake level of examined As and toxic metals were lower than the level of concern (HQ < 1); therefore, the non-carcinogenic risk from heavy metals via ingestion of drinking water was in the safe range for children and adult population. Finding from our study is in agreement with results of other studies in Malaysia and Pakistan [2, 39].

In the case of the dermal pathway, the HQ and HI values never exceeded the level of concern for adult, while the HI for children was higher than the threshold of HI at stations 7, 17, 23, and 34.

This result showed that children suffered more adverse health risk through dermal contact with water due to their higher skin adherence compared to adults. As the result showed, the HQ_dermal contact_ of Cr determined for more than 63% of HI_dermal contact_ for children. This was consistent with the previous studies; they reported that dermal contact with Cr contributed to higher non-carcinogenic risk compared to other exposure routes [17, 40].

Furthermore, Cr showed the highest average contribution of HI_total elements_ (55 to 71.2%) for adult and child population (Fig. 3). It seems Cr could be the most hazardous element in the case of non-carcinogen risk. Notably, the Cr^6+^ is much more toxic than Cr^3+^ and other metals that were used to assess human exposure in this study. However, Cr^6+^ is decreased into Cr^3+^ in the human body; thus, there might be an overestimation in determining health risk [17, 41]. It should be noted that the HI_total elements_ (HQ_ingestion_ + HQ_dermal contact_) values for children were higher than that of an adult, suggesting that children were more susceptible to non-carcinogenic risk from the heavy metals. This result is in agreement with the results reported in Australia [10] and the Hong Kong Environmental Protection Department [1].

#### Carcinogenic risk

The cancer risk was determined based on the intake level of inorganic As, Pb and Cr, which may increase carcinogenic effects depending on the exposure dose [33, 42]. Briefly, based on dermal exposure, the chance of developing CR_dermal contact_ for all elements ranged from 9.62 × 10^−7^ to 8.72 × 10^−5^, and its average values for children and adults were 3. 4 × 10^−5^ and 6.42 × 10^−6^, respectively. Thus, the CR_dermal contact_ values were below the safety level (1 × 10^−4^) recommended by the US EPA, suggesting carcinogenic risk can be acceptable for both adult and children in Mashhad through dermal contact (Additional file 1: Table S2).

Considering ingestion exposure pathways, estimated CR_ingestion_ for all elements was in the range of 1.65 × 10^−6^ to 8.05 × 10^−5^ for adults and in 5.07 × 10^−6^to 2.84 × 10^−4^ for children (Additional file 1: Table S2). The average values of CR_ingestion_ for adults and children were 4.38 × 10^−5^ and 1.27 × 10^−4^, respectively. This suggested that the probability of carcinogenic risk for children via the consumption of drinking water collected from Mashhad was 1.27 in 10000, while for adult was 4.38 in 100,000, indicating the potential CR_ingestion_ for the children population from lifetime exposure to the carcinogenic elements (As, Pb, and Cr) via ingestion of drinking water in Mashhad, Iran. The study also found that Cr had the highest average contribution of TCR (63.2%) compared to other carcinogenic elements such as Pb (24.0%) and As (15.7%) (Table 7). It seems that Cr could be the most hazardous element in the case of carcinogen risk.

**Table 7:** Descriptive statistics of total carcinogenic risk (carcinogenic risk through ingestion and dermal combined) for children and adults

Sites/stations (*n* = 140)		Carcinogenic risk (CR)							
		As		Pb		Cr		Total CR	
		Adult	Child	Adult	Child	Adult	Child	Adult	Child
North	1	7.98E−06	1.71E−05	2.5E−05	4.03E−05	3.81E−06	6.63E−06	3.77E−05	6.41E−05
	2	7.44E−06	1.60E−05	1.2E−05	2.29E−05	5.18E−06	9.02E−06	2.56E−05	4.79E−05
	3	6.38E−06	1.37E−05	2.2E−05	3.39E−05	8.90E−06	1.55E−05	3.73E−05	6.31E−05
	4	1.44E−05	3.09E−05	3.3E−05	5.50E−05	5.53E−05	9.63E−05	1.03E−04	1.82E−04
	5	7.98E−06	1.71E−05	1.5E−05	2.62E−05	2.50E−05	4.35E−05	4.81E−05	8.68E−05
	6	1.33E−05	2.46E−05	2.2E−05	3.04E−05	3.35E−05	5.03E−05	6.88E−05	1.26E−04
	7	1.06E−05	2.29E−05	1.1E−05	2.34E−05	1.62E−04	2.82E−04	1.84E−04	3.28E−04
South	8	9.04E−06	1.94E−05	1.1E−05	2.19E−05	3.30E−06	5.75E−06	2.36E−05	4.71E−05
	9	4.79E−06	1.03E−05	1.6E−05	2.53E−05	4.91E−06	8.54E−06	2.61E−05	4.41E−05
	10	1.06E−05	2.29E−05	1.5E−05	2.03E−05	3.51E−05	5.11E−05	6.09E−05	1.12E−04
	11	1.12E−05	2.40E−05	1.2E−05	2.46E−05	7.57E−05	1.32E−04	9.88E−05	1.80E−04
	12	9.57E−06	2.06E−05	9.6E−06	2.03E−05	7.58E−05	1.32E−04	9.51E−05	1.73E−04
	13	7.44E−06	1.60E−05	1.8E−05	3.01E−05	5.88E−05	1.02E−04	8.47E−05	1.48E−04
	14	9.57E−06	2.06E−05	1.2E−05	2.37E−05	9.37E−05	1.63E−04	1.16E−04	2.07E−04
East	15	5.32E−06	1.14E−05	1.0E−05	1.77E−05	6.24E−06	1.09E−05	2.18E−05	4.00E−05
	16	1.01E−05	2.17E−05	1.6E−05	2.91E−05	1.02E−04	1.78E−04	1.29E−04	2.29E−04
	17	6.38E−06	1.37E−05	1.1E−05	2.04E−05	2.13E−04	3.71E−04	2.31E−04	4.05E−04
	18	1.49E−05	3.20E−05	1.9E−05	3.74E−05	4.31E−06	7.50E−06	3.87E−05	7.69E−05
	19	1.12E−05	2.40E−05	1.5E−05	2.91E−05	5.18E−06	9.02E−06	3.18E−05	6.21E−05
	20	7.98E−06	1.71E−05	1.7E−05	2.93E−05	5.41E−06	9.42E−06	3.08E−05	5.58E−05
	21	8.51E−06	1.43E−05	2.3E−05	3.75E−05	3.52E−05	5.24E−05	7.01E−05	1.22E−04
West	22	8.51E−06	1.83E−05	1.6E−05	2.89E−05	2.22E−05	3.86E−05	4.75E−05	8.57E−05
	23	2.07E−05	4.46E−05	4.1E−05	7.09E−05	5.82E−05	1.01E−04	1.20E−04	2.17E−04
	24	9.57E−06	2.06E−05	9.6E−06	2.03E−05	6.89E−05	5.20E−05	8.81E−05	1.61E−04
	25	9.57E−06	2.06E−05	1.9E−05	3.32E−05	2.89E−06	5.03E−06	3.19E−05	5.88E−05
	26	1.22E−05	2.63E−05	1.6E−05	3.14E−05	1.09E−05	1.89E−05	3.96E−05	7.66E−05
	27	1.28E−05	2.74E−05	2.0E−05	3.63E−05	4.09E−05	5.11E−05	7.36E−05	1.35E−04
	28	1.06E−05	2.29E−05	2.3E−05	3.89E−05	5.10E−05	6.88E−05	8.49E−05	1.51E−04
Center	29	1.22E−05	2.63E−05	1.5E−05	3.04E−05	3.74E−05	6.51E−05	6.54E−05	1.22E−04
	30	1.06E−05	2.29E−05	1.6E−05	3.05E−05	2.32E−05	4.03E−05	5.06E−05	9.37E−05
	31	7.44E−06	1.60E−05	1.7E−05	2.88E−05	1.46E−05	2.54E−05	3.95E−05	7.02E−05
	32	8.51E−06	1.83E−05	1.7E−05	3.03E−05	7.23E−05	1.26E−04	9.87E−05	1.74E−04
	33	6.91E−06	1.49E−05	1.4E−05	2.39E−05	1.32E−05	2.29E−05	3.41E−05	6.17E−05
	34	1.06E−05	2.29E−05	2.5E−05	4.16E−05	1.79E−04	3.11E−04	2.15E−04	3.76E−04
	35	7.44E−06	1.60E−05	1.9E−05	3.19E−05	3.67E−06	6.39E−06	3.09E−05	5.43E−05
Mean		9.78E−06	2.10E−05	1.7E−05	3.12E−05	4.61E−05	8.03E−05	7.38E−05	1.33E−04
Standard deviation		3.07E−06	6.61E−06	6.5E−06	1.03E−05	5.20E−05	9.05E−05	5.25E−05	9.20E−05

**Fig. 4:**
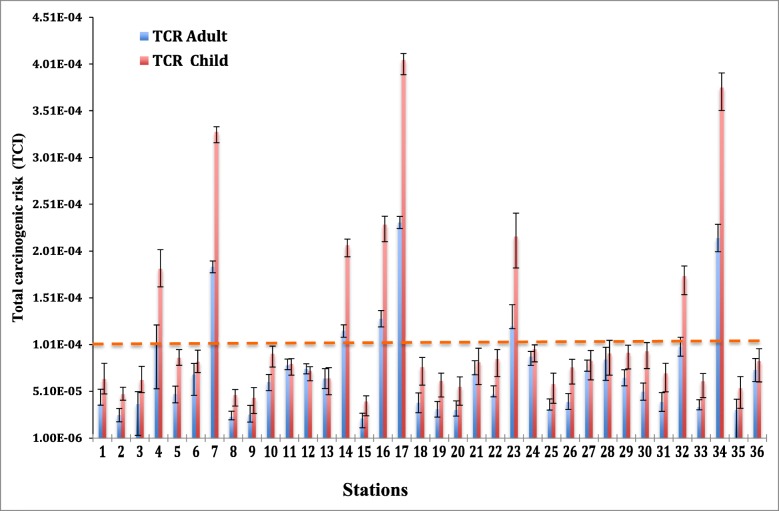
Estimated total carcinogenic risk (total) for drinking water through ingestion and dermal contact by adult and children

Based on the total CR (TCR = CR_dermal contact_ + CR_ingestion_) values, the chance of developing CR ranged from 2.36 × 10^−5^ to 3.76 × 10^−4^, and its average values for children and adults were 1.33 × 10^−4^ and 7.38 × 10^−5^, respectively (Table 7 and Fig. 4). This result confirmed a potential cancer risk for the children as a highly exposed population to the carcinogenic elements via ingestion and dermal routes, particularly at stations 4, 7, 14, 16, 17, 23, 32, and 34. However, a potential TCR was borderline (1 × 10^−4^ to 1 × 10^−6^ ) for the adult population. Therefore, the consumption of drinking water in this area could be large enough to warrant action under Superfund guidelines and may pose detrimental health hazards to the exposed population [1, 9, 10]. With regard to the different pathways, the contribution of dermal exposure was lower than (40.2% for adult and 32.4% for children) digestion exposure to the TCR, which is in consistence with more recent studies [1, 10].

It is noteworthy to indicate that the estimated TCR was higher for children compared to adults, suggesting that children were more susceptible to CR from the As and heavy metals. According to the recent World Health Organization (WHO) report, children are a vulnerable population to health risks because they drink more water, consume more food, and breathe more air in proportion to their weight. Children’s immune, digestive, reproductive, and nervous systems are still growing. At the early part of development, exposure to toxic elements causes irreversible damage [43]. According to the finding, all stations require some intervention, remediation, and control measures to decrease the level of carcinogenic heavy metals. It is suggested that appropriate purification improvement programs should be implemented to protect the health of the residents in this metropolitan city, especially from stations 4, 7, 14, 16, 17, 23, 32, and 34.

#### Uncertainty of risk

In this work, there is the possibility of uncertainties that may not be taken into account and could consider as a limitation for the validity of the risk estimation. For instance, (i) body weights and daily intake of drinking water were not estimated for the people who live in Mashhad, (ii) most of the probability variables applied for estimation were derived from the US EPA guideline which may not apply to this population, (iii) CSF of As and Pb was only used to assess CR because there is no real CSF value available for other toxic metals, (iv) CSF was considered as a constant for all individuals, but in reality, CSF can change between individuals, and (v) the health risk was only assessed using the heavy metal toxicity, but the fact is that drinking water also contains other chemicals from possible exposure. Thus, the level of risk from drinking water in Mashhad may be higher than that estimated values in this work.

## Conclusion

In this study, the health risk assessment of heavy metals was evaluated based on daily intake and exposure through dermal absorption and ingestion of drinking water of the two selected populations of adults and children. Likewise, we focused on two populations for health risks assessment, including adult and children (as a sensitive population). Drinking water ingestion was the main metal exposure routes for Mashhad residents, followed by dermal contact pathway. For both target groups, the daily heavy metal intakes via water consumption were at least four to ten times higher than those via dermal contact. For dermal exposure, the non-carcinogenic and carcinogenic risk level for arsenic and heavy metals never exceeded the US EPA risk management criterion, suggesting there is no health risk threat from heavy metals for adults and children. However, risk evaluation showed that for children at stations 7, 17, 23, 3and 4, there is non-carcinogenic risk via dermal contact. Risk evaluation indicated that the carcinogenic risk from the consumption of drinking water based on ingestion exposure was borderline or higher than the safety level of US EPA risk; therefore, residents in this study area might suffer more health risk and serious attention must be given in this area. The exposure assessments exhibited children might suffer more carcinogenic and non-carcinogen risk via ingestion and dermal contact routes, and residents in Mashhad was more exposed to Cr. Likewise, the uncertainty of risk explained major variables for the probabilistic health risk determination; therefore, health risk via consumption of drinking water could be higher than that estimated values. More efforts are needed to reduce the heavy metal level in drinking water in Mashhad, such as appropriate purification system and the control of the heavy metal discharge. Furthermore, proper use of wastewater treatment plants must be implemented to protect the local population and reduce human health risks.

## Additional file

Additional file 1: **Table S1.** ICP-MS operating measurement. **Table S2.** Descriptive statistics of total carcinogenic risk (carcinogenic risk through ingestion and dermal combined) for children and adult. (DOCX 28 kb)

## Data Availability

The datasets generated and analyzed during this study are included in the main document of this manuscript.

## Abbreviations

ADD: Average daily dose
AT: Averaging time
BW: Body weight
CF: Conversion factor
EC: Electrical conductivity
ED: Exposure duration
EF: Exposure frequency
HHRA: Human health risk assessment
HI: Hazard index
HQ: Hazard quotients
IARC: Agency for Research on Cancer
IR: Ingestion rate
LOD: Limit of detection
RFD: Reference dose
SA: Skin surface
TCR: Total carcinogenic risk
THQ: Target hazard quotient
US EPA: United States Environmental Protection Agency
WHO: World Health Organization
