# Supplementary material for: Health risk assessments of arsenic and toxic heavy metal exposure in drinking water in northeast Iran
Source: Environ Health Prev Med. 2019 Sep 14;24:59. doi: 10.1186/s12199-019-0812-x (PMC6745075; doi:10.1186/s12199-019-0812-x)
Supplement: Supplementary file 1 — Table S1. ICP-MS operating measurement. Table S2. Descriptive statistics of total carcinogenic risk (carcinogenic risk through ingestion and dermal combined) for children and adult. (DOCX 28 kb) [file 12199_2019_812_MOESM1_ESM.docx]

Table S1. ICP-MS operating measurement

| **Operating condition** |  |
| --- | --- |
| Spray chamber | Scott double-pass |
| RF power (W) | 1550 |
| Nebuliser pump (rps) | 0.1 |
| Sample depth (mm) | 8 |
| RF Matching (V) | 0.2 |
| Plasma gas flow rate (l min^−1^) | 15 |
| Torch-V (mm) | 0.2 |
| Carrier gas (Ar) flow rate (l min^−1^) | 0.9 (optimized daily) |
| **Measurement Factores** |  |
| Scanning mode | Peak hop |
| Readings/replicate | 1 |
| Resolution (amu) | 0.7 |
| Number of replicates | 3 |

Table S2: Descriptive statistics of total carcinogenic risk (carcinogenic risk through ingestion and

dermal combined) for children and adult

|  | As | | | | Pb | | | | Cr | | | |
| --- | --- | --- | --- | --- | --- | --- | --- | --- | --- | --- | --- | --- |
|  | Ingestion | | Dermal | | Dermal | | Ingestion | | Dermal | | Ingestion | |
|  | **Adult** | **Child** | **Adult** | **Child** | **Adult** | **child** | **Adult** | **Child** | **Adult** | **Child** | **Adult** | **Child** |
| **Stations** | 2.8E-06 | 1.1E-05 | 5.2E-06 | 6.3E-06 | 8.6E-06 | 3.4E-05 | 1.7E-05 | 2.1E-05 | 2.5E-06 | 5.1E-06 | 1.3E-06 | 1.6E-06 |
| 1 | 2.6E-06 | 1.0E-05 | 4.9E-06 | 5.9E-06 | 4.3E-06 | 1.7E-05 | 8.6E-06 | 1.1E-05 | 3.5E-06 | 6.9E-06 | 1.7E-06 | 2.1E-06 |
| 2 | 2.2E-06 | 8.7E-06 | 4.2E-06 | 5.0E-06 | 7.3E-06 | 2.9E-05 | 1.5E-05 | 1.8E-05 | 5.9E-06 | 1.2E-05 | 3.0E-06 | 3.6E-06 |
| 3 | 5.0E-06 | 2.0E-05 | 9.4E-06 | 1.1E-05 | 1.1E-05 | 4.4E-05 | 2.2E-05 | 2.7E-05 | 3.7E-05 | 7.4E-05 | 1.8E-05 | 2.3E-05 |
| 4 | 2.8E-06 | 1.1E-05 | 5.2E-06 | 6.3E-06 | 5.0E-06 | 2.0E-05 | 1.0E-05 | 1.2E-05 | 1.7E-05 | 3.3E-05 | 8.3E-06 | 1.0E-05 |
| 5 | 4.6E-06 | 1.8E-05 | 8.7E-06 | 1.1E-05 | 7.3E-06 | 2.9E-05 | 1.5E-05 | 1.8E-05 | 2.2E-05 | 4.5E-05 | 1.1E-05 | 1.4E-05 |
| 6 | 3.7E-06 | 1.4E-05 | 7.0E-06 | 8.4E-06 | 3.8E-06 | 1.5E-05 | 7.6E-06 | 9.3E-06 | 1.1E-04 | 2.2E-04 | 5.4E-05 | 6.6E-05 |
| 7 | 3.1E-06 | 1.2E-05 | 5.9E-06 | 7.1E-06 | 3.7E-06 | 1.5E-05 | 7.5E-06 | 9.2E-06 | 2.2E-06 | 4.4E-06 | 1.1E-06 | 1.4E-06 |
| 8 | 1.7E-06 | 6.5E-06 | 3.1E-06 | 3.8E-06 | 5.5E-06 | 2.2E-05 | 1.1E-05 | 1.3E-05 | 3.3E-06 | 6.5E-06 | 1.6E-06 | 2.0E-06 |
| 9 | 3.7E-06 | 1.4E-05 | 7.0E-06 | 8.4E-06 | 5.0E-06 | 2.0E-05 | 1.0E-05 | 1.2E-05 | 2.3E-05 | 4.7E-05 | 1.2E-05 | 1.4E-05 |
| 10 | 3.9E-06 | 1.5E-05 | 7.3E-06 | 8.8E-06 | 4.0E-06 | 1.6E-05 | 8.0E-06 | 9.8E-06 | 5.0E-05 | 1.0E-04 | 2.5E-05 | 3.1E-05 |
| 11 | 3.3E-06 | 1.3E-05 | 6.3E-06 | 7.6E-06 | 3.2E-06 | 1.3E-05 | 6.4E-06 | 7.9E-06 | 5.1E-05 | 1.0E-04 | 2.5E-05 | 3.1E-05 |
| 12 | 2.6E-06 | 1.0E-05 | 4.9E-06 | 5.9E-06 | 6.1E-06 | 2.4E-05 | 1.2E-05 | 1.5E-05 | 3.9E-05 | 7.8E-05 | 2.0E-05 | 2.4E-05 |
| 13 | 3.3E-06 | 1.3E-05 | 6.3E-06 | 7.6E-06 | 4.1E-06 | 1.6E-05 | 8.2E-06 | 1.0E-05 | 6.3E-05 | 1.2E-04 | 3.1E-05 | 3.8E-05 |
| 14 | 1.8E-06 | 7.2E-06 | 3.5E-06 | 4.2E-06 | 3.4E-06 | 1.4E-05 | 6.9E-06 | 8.4E-06 | 4.2E-06 | 8.3E-06 | 2.1E-06 | 2.6E-06 |
| 15 | 3.5E-06 | 1.4E-05 | 6.6E-06 | 8.0E-06 | 5.4E-06 | 2.1E-05 | 1.1E-05 | 1.3E-05 | 6.8E-05 | 1.4E-04 | 3.4E-05 | 4.2E-05 |
| 16 | 2.2E-06 | 8.7E-06 | 4.2E-06 | 5.0E-06 | 3.9E-06 | 1.5E-05 | 7.8E-06 | 9.6E-06 | 1.4E-04 | 2.8E-04 | 7.1E-05 | 8.7E-05 |
| 17 | 5.1E-06 | 2.0E-05 | 9.7E-06 | 1.2E-05 | 6.5E-06 | 2.6E-05 | 1.3E-05 | 1.6E-05 | 2.9E-06 | 5.7E-06 | 1.4E-06 | 1.8E-06 |
| 18 | 3.9E-06 | 1.5E-05 | 7.3E-06 | 8.8E-06 | 5.2E-06 | 2.0E-05 | 1.0E-05 | 1.3E-05 | 3.5E-06 | 6.9E-06 | 1.7E-06 | 2.1E-06 |
| 19 | 2.8E-06 | 1.1E-05 | 5.2E-06 | 6.3E-06 | 5.8E-06 | 2.3E-05 | 1.2E-05 | 1.4E-05 | 3.6E-06 | 7.2E-06 | 1.8E-06 | 2.2E-06 |
| 20 | 2.9E-06 | 1.2E-05 | 5.6E-06 | 6.7E-06 | 7.8E-06 | 3.1E-05 | 1.6E-05 | 1.9E-05 | 2.5E-05 | 5.1E-05 | 1.3E-05 | 1.6E-05 |
| 21 | 2.9E-06 | 1.2E-05 | 5.6E-06 | 6.7E-06 | 5.6E-06 | 2.2E-05 | 1.1E-05 | 1.4E-05 | 1.5E-05 | 2.9E-05 | 7.4E-06 | 9.1E-06 |
| 22 | 7.2E-06 | 2.8E-05 | 1.4E-05 | 1.6E-05 | 1.4E-05 | 5.5E-05 | 2.8E-05 | 3.4E-05 | 3.9E-05 | 7.7E-05 | 1.9E-05 | 2.4E-05 |
| 23 | 3.3E-06 | 1.3E-05 | 6.3E-06 | 7.6E-06 | 3.2E-06 | 1.3E-05 | 6.4E-06 | 7.9E-06 | 4.6E-05 | 9.2E-05 | 2.3E-05 | 2.8E-05 |
| 24 | 3.3E-06 | 1.3E-05 | 6.3E-06 | 7.6E-06 | 6.5E-06 | 2.6E-05 | 1.3E-05 | 1.6E-05 | 1.9E-06 | 3.8E-06 | 9.6E-07 | 1.2E-06 |
| 25 | 4.2E-06 | 1.7E-05 | 8.0E-06 | 9.7E-06 | 5.5E-06 | 2.2E-05 | 1.1E-05 | 1.4E-05 | 7.3E-06 | 1.4E-05 | 3.6E-06 | 4.4E-06 |
| 26 | 4.4E-06 | 1.7E-05 | 8.4E-06 | 1.0E-05 | 6.7E-06 | 2.6E-05 | 1.3E-05 | 1.6E-05 | 2.7E-05 | 5.4E-05 | 1.4E-05 | 1.7E-05 |
| 27 | 3.7E-06 | 1.4E-05 | 7.0E-06 | 8.4E-06 | 7.8E-06 | 3.1E-05 | 1.5E-05 | 1.9E-05 | 3.4E-05 | 6.8E-05 | 1.7E-05 | 2.1E-05 |
| 28 | 4.2E-06 | 1.7E-05 | 8.0E-06 | 9.7E-06 | 5.3E-06 | 2.1E-05 | 1.0E-05 | 1.3E-05 | 2.5E-05 | 5.0E-05 | 1.2E-05 | 1.5E-05 |
| 29 | 3.7E-06 | 1.4E-05 | 7.0E-06 | 8.4E-06 | 5.6E-06 | 2.2E-05 | 1.1E-05 | 1.4E-05 | 1.5E-05 | 3.1E-05 | 7.7E-06 | 9.5E-06 |
| 30 | 2.6E-06 | 1.0E-05 | 4.9E-06 | 5.9E-06 | 5.8E-06 | 2.3E-05 | 1.2E-05 | 1.4E-05 | 9.7E-06 | 1.9E-05 | 4.9E-06 | 6.0E-06 |
| 31 | 2.9E-06 | 1.2E-05 | 5.6E-06 | 6.7E-06 | 6.0E-06 | 2.4E-05 | 1.2E-05 | 1.5E-05 | 4.8E-05 | 9.6E-05 | 2.4E-05 | 3.0E-05 |
| 32 | 2.4E-06 | 9.4E-06 | 4.5E-06 | 5.5E-06 | 4.7E-06 | 1.8E-05 | 9.3E-06 | 1.1E-05 | 8.8E-06 | 1.8E-05 | 4.4E-06 | 5.4E-06 |
| 33 | 3.7E-06 | 1.4E-05 | 7.0E-06 | 8.4E-06 | 8.4E-06 | 3.3E-05 | 1.7E-05 | 2.1E-05 | 1.2E-04 | 2.4E-04 | 6.0E-05 | 7.3E-05 |
| 34 | 2.6E-06 | 1.0E-05 | 4.9E-06 | 5.9E-06 | 6.6E-06 | 2.6E-05 | 1.3E-05 | 1.6E-05 | 2.4E-06 | 4.9E-06 | 1.2E-06 | 1.5E-06 |
| 35 | 3.4E-06 | 1.3E-05 | 6.4E-06 | 7.7E-06 | 6.0E-06 | 2.3E-05 | 1.2E-05 | 1.5E-05 | 3.1E-05 | 6.1E-05 | 1.5E-05 | 1.9E-05 |
